# Anodized Titanium Implant Abutments: Effects on Surface Properties, Peri-Implant Soft Tissue Esthetics, and Biological Outcomes—A Focused Narrative Review

**DOI:** 10.3390/dj14070403

**Published:** 2026-07-03

**Authors:** Elana Y. Laks, Amal Al-Faraj, Chao-Chieh Yang, Michele L. Kirkup, John A. Levon, Wei-Shao Lin

**Affiliations:** Department of Prosthodontics, Indiana University School of Dentistry, Indianapolis, IN 46202, USA

**Keywords:** anodization, anodic oxidation, titanium abutments, dental implants, peri-implant mucosa, esthetics, soft tissue thickness, hydrophilicity, biofilm

## Abstract

**Background/Objectives:** To summarize evidence on whether anodization of titanium implant abutments modifies abutment surface/physical properties, peri-implant soft tissue esthetics, and biological outcomes compared with machined titanium and alternative abutment materials. **Methods:** This focused narrative review used a structured, reproducible literature search with dual-reviewer screening and data extraction. The study selection pathway was summarized using a PRISMA-style flow diagram for transparency. Searches of PubMed/MEDLINE, EMBASE, CENTRAL, and Google Scholar were performed, supplemented by gray literature screening and manual searching. Laboratory, animal, and human clinical studies evaluating anodized titanium abutments were eligible. Data were synthesized qualitatively across prespecified domains (surface or physical properties, esthetic outcomes, and biological outcomes). Because the work was designed as a narrative review, protocol registration and formal risk of bias appraisal were not undertaken. **Results:** Thirty-five studies were included (twenty-five in vitro, three animal, and seven human clinical). Anodization modified oxide layer characteristics and surface chemistry, and was commonly associated with increased hydrophilicity. Esthetically, pink or yellow anodized titanium generally reduced discoloration compared with uncolored titanium, particularly when soft tissue and restorative material thickness were sufficient, whereas zirconia most consistently produced the most favorable color outcomes. In vitro studies frequently reported improved early soft tissue cell responses and, in selected protocols, reduced bacterial adhesion. Reported clinical differences in inflammation indices and peri-implant marginal bone changes were small or inconsistent. **Conclusions:** Anodization can improve the optical masking of titanium and alter surface wettability and chemistry, but evidence for sustained clinical biological benefit remains limited. Current evidence supports anodization primarily as an esthetic adjunct, while long-term peri-implant biological outcomes appear to be driven by multiple clinical factors beyond surface modification.

## 1. Introduction

Titanium implant abutments are commonly used due to their biocompatibility, strength, and predictable clinical performance. However, their inherent gray color can negatively affect esthetic outcomes, especially in sites with a thin gingival phenotype. Several surface modifications have been developed to improve the appearance of titanium, including anodization (anodic oxidation), which specifically targets this limitation [1,2,3,4,5,6].

Anodization is an electrolytic surface modification technique in which the metal’s natural oxide layer is thickened (approximately 30–150 nm) by applying specific voltages in an electrolytic solution [7,8]. The titanium component functions as the anode, and the applied current promotes oxide growth [9,10]. The resulting thickened oxide layer can display different colors due to light interference, which can improve esthetic outcomes [11,12]. Thus, titanium implant abutments are generally anodized to display a pink or gold color to better blend with peri-implant tissues and reduce the gray appearance [13,14,15].

In addition to aesthetic changes, anodization can modify the titanium’s physical properties, such as surface roughness, wettability, oxide layer thickness, and surface topography [5,6,8]. These physical modifications may subsequently influence biological outcomes at the implant-abutment interface. Some clinical studies have reported that anodized abutments may lower the bleeding index at abutment removal and may increase the height of the keratinized mucosa. It is important to establish an adequate band of keratinized mucosa around the implants, as insufficient height may be associated with greater soft tissue discomfort during hygiene [16,17]. Additionally, other research suggests that anodic oxidation of titanium may even improve corrosion resistance and durability because of the metal’s increased oxide-layer thickness [18,19,20]. However, it is shown that anodic oxidation performed at lower temperatures results in oxide layers with higher hardness [18]. Because anodization influences esthetic, physical, and biological parameters, a thorough review of the existing literature is needed to clarify its clinical significance.

The aim of this study was to conduct a narrative review, supported by a structured study-identification and selection process, to evaluate the effects of anodized titanium abutments compared with machined titanium and alternative abutment materials on (1) abutment surface/physical properties, (2) peri-implant soft tissue esthetics, and (3) biological outcomes.

## 2. Materials and Methods

This focused narrative review was conducted using a structured literature search and predefined eligibility criteria, and the study selection pathway was summarized using a PRISMA-style flow diagram to enhance transparency. Because this was a narrative (qualitative) synthesis, no meta-analysis was planned. The focused question was formulated using the PICO (Population, Intervention, Comparison, Outcome) format: “For patients receiving dental implants, does anodization of titanium abutments, compared with machined titanium or alternative abutment materials, improve (1) abutment surface or physical properties, (2) peri-implant soft tissue esthetics, and (3) biological outcomes?” The review was not prospectively registered, and a protocol was not prepared.

Studies were included if they were laboratory, animal, or human clinical investigations published between 2002 and 2025 that evaluated anodized titanium implant abutments (or transmucosal abutment surfaces) and reported outcomes relevant to at least one of the three prespecified domains (surface/physical, esthetic, and/or biological). Studies were excluded if they were methodology/technique papers or review articles, involved duplicate publications from the same dataset, lacked identifiable information relevant to implant abutments or transmucosal components, did not report extractable outcomes aligned with the review question, or were published in a non-English language.

The review search strategy was conducted electronically on PubMed/MEDLINE, Cochrane Central Register of Controlled Trials (CENTRAL), EMBASE, and Google Scholar by using MeSH terms and keywords associated with the focused question (Table 1). Gray literature was searched through electronic screening using the New York Academy of Medicine Grey Literature report (greylit.org) and through Google Scholar. All records were imported into a review management software (Covidence; Melbourne, Australia) to aid data collection and extraction. Additionally, manual searching was conducted in Clinical Oral Implants Research, International Journal of Oral Maxillofacial Implants, Clinical Implant Dentistry and Related Research, Journal of Oral Implantology, Implant Dentistry, Journal of Prosthetic Dentistry, Journal of Prosthodontics, and International Journal of Prosthodontics, as well as relevant dental materials journals and personal communications identified through gray literature. The final electronic search was completed on 15 January 2026.

Two reviewers independently screened titles/abstracts and full texts to determine eligibility, and any difference in opinion was resolved via personal discussion and consensus. Regarding data extraction, the following information was extracted and collected from the selected studies: general study characteristics (authors, publication year, types of study, sample size, types of implants, types of abutments, anodization methods), physical properties (Scanning Electron Microscopy [SEM] imaging, X-ray diffractometry [XRD], chemical elemental analysis, surface characteristics [Sa, Sq, Sz, Sdr, Sdq, Sal, Str], and water contact angle [WCA] measurement), esthetic outcomes (color difference [ΔL*, Δa*, Δb*, ΔE] among abutments), and biological properties (soft tissue outcomes, radiographic analysis, biocompatibility, and histometric analysis). Results were summarized in tables and synthesized qualitatively within the three outcome domains rather than by study model.

## 3. Results

### 3.1. Study Screening and Selection

**Figure 1:**
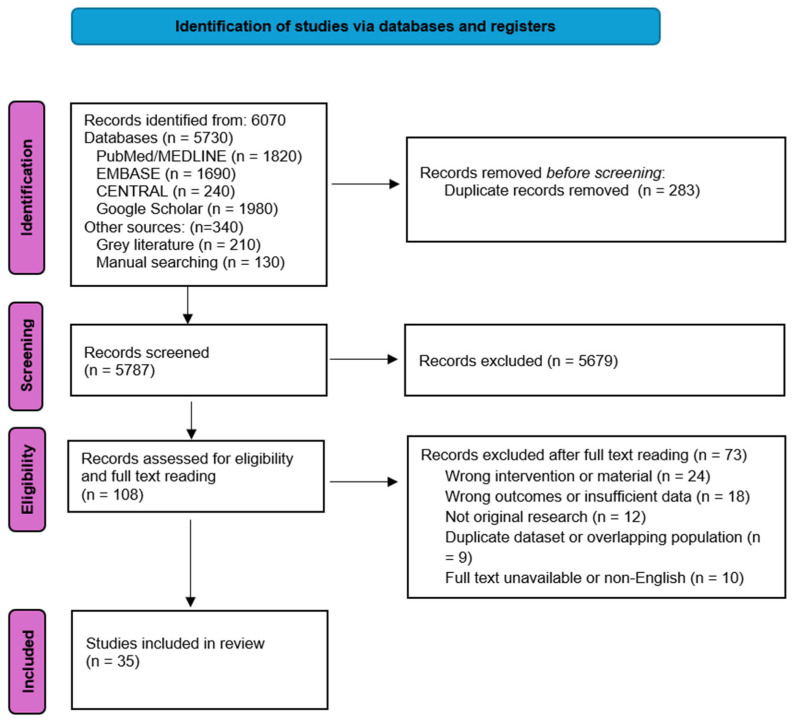
Study selection flow diagram (PRISMA-style) for records identified, screened, and included.

After the initial electronic and manual search, 6070 studies were found. After removing 283 duplicate studies, 5787 studies were included in the initial screening. After title and abstract screening, 5679 studies were excluded as they did not meet the inclusion criteria. A total of 108 studies were included in the full-text review, while 73 studies were excluded. A total of 35 studies were included in the review. The included evidence comprised 25 in vitro studies, 3 animal studies, and 7 human clinical studies . Two reviewers (E.Y.L. and A.A.) independently assessed the full texts to determine eligibility (inclusion/exclusion), and disagreements were resolved by discussion and consensus. The PRISMA flow diagram visually summarizes the inclusion process (Figure 1).

### 3.2. Data Extraction and Analysis

Data extraction and qualitative comparisons were completed from the 35 included studies. The summarized results can be found in Table 2 and Table 3.

### 3.3. Physical Properties

#### 3.3.1. Scanning Electron Microscopy (SEM) Imaging

SEM was used to evaluate how anodization changes the titanium surface topography and its effect on the surrounding soft tissue. Multiple studies demonstrated how an increase in voltage during the anodization process produced a more organized nanotube structure, with 80 V showing the highest level of soft tissue integration. Some authors reported micro-cracks and grain formation in the oxide layer, which may be a result of oxygen bubble formation occurring during the anodization process. This may suggest possible changes within the oxide layer itself during the anodization process, not just changes in the surface topography [23].

Increased change in surface topography was reported in studies where roughening techniques were used, whereas minimal change was reported from just the anodization process alone [21,30]. SEM analysis showed increased fibroblast and osteoblast attachment on anodized surfaces compared with smooth machined surfaces, and smaller pore diameters were associated with reduced biofilm formation [22,27,36]. Additionally, biofilm imaging was obtained with SEM and showed that an atomic-layer-deposited coating resisted bacteria due to its smooth and dense surface, whereas porous anodized surfaces did not consistently reduce bacterial attachment [31]. Across studies, SEM was used both to confirm surface morphology/material identity and to visualize cell adhesion and morphology [24,25,26,28,29,31,33].

#### 3.3.2. X-Ray Diffractometry (XRD)

XRD was used by Mendez et al. and Mühl et al. to identify the crystalline phases of the oxide layer after anodization [29,36].

#### 3.3.3. Chemical Elemental Analysis

Chemical elemental analyses generally reported higher oxygen signals after anodization; however, these findings are best interpreted as reflecting growth and thickening of the TiO_2_ layer within the analytical sampling depth, rather than true oxygen enrichment of the outermost titanium dioxide surface. Such oxide-layer growth contributes to the interference colors observed after anodization [7,21,22,23,24,30,31].

#### 3.3.4. Surface Characteristics

Surface characteristic measurements frequently showed lower contact-angle values after anodization, suggesting increased hydrophilicity under the specific tested conditions and, in some protocols, altered roughness, which may contribute to increased fibroblast attachment [23,25,26,29,36,37].

#### 3.3.5. Water Contact Angle (WCA)

Most of the papers found that anodization lowered the WCA and increased hydrophilicity, which may support early soft tissue healing. However, WCA measurements should be interpreted cautiously because they can be influenced by cleaning and storage conditions, surface roughness, adventitious contamination, liquid type, and the time elapsed after surface preparation. This trend applied up to approximately 60 V, while higher applied voltages resulted in irregular oxide layers with decreased hydrophilicity [7,21,22,23,25,26,28,29,30,33,36,37]. Additional reported physical effects included increased tensile bond strength between lithium disilicate crowns and titanium abutments after anodization, in some studies without substantial change in surface topography [32].

### 3.4. Esthetic Outcomes

Most studies reported that zirconia abutments displayed the lowest ΔE values and produced superior esthetic outcomes. However, yellow- or pink-anodized titanium abutments often improved esthetic outcomes compared with uncolored titanium, particularly when ≥2 mm of ceramic or soft tissue was present. Overall, anodization tended to reduce soft tissue discoloration relative to unmodified titanium, although zirconia most consistently demonstrated the most favorable color outcomes across conditions [7,8,14,16,21,40]. More opaque, lighter-colored cements also contributed to reduced ΔE values [7,8,14,16,34,38,40,41,42,43,44,45,46,47,49].

### 3.5. Biological Properties

#### 3.5.1. Soft Tissue Outcomes

Across the in vitro studies the material that consistently displayed the best fibroblast proliferation and attachment was zirconia. However, anodized titanium displayed increased fibroblast alignment, viability, and adhesion compared with machined titanium. Additionally, anodized titanium with nanoporous surfaces displayed improved tissue sealing and soft tissue outcomes in several laboratory models, and reduced bacterial adhesion was reported in some protocols [22,23,24,25,26,27,28,29,30,31,33,36]. In animal and clinical studies, reported differences between anodized and machined titanium were generally small and not consistently significant, and factors such as abutment geometry (e.g., slim versus regular) and soft tissue thickness may have a greater influence on soft tissue outcomes [8,14,16,17,35,37,39,48,49].

#### 3.5.2. Radiographic Analysis

Across most studies, radiographic analysis showed that there was no significant difference between implant stability and peri-implant marginal bone remodeling associated with the anodized titanium abutments [8,14,16,17,27,30,37,39,40,48,49].

#### 3.5.3. Biocompatibility

Across most studies, zirconia was frequently reported as highly biocompatible, and anodized titanium generally performed similarly to conventional titanium with respect to fibroblast adhesion and proliferation, with some protocols reporting reduced bacterial adhesion and improved early soft tissue healing markers. Some studies reported improved mechanics associated with the machined abutments; however, the anodized abutments have consistently shown to be compatible in the esthetic zone with superior esthetics and soft tissue integration [7,8,14,16,17,21,23,27,30,31,33,35,37,39,48,49,50].

#### 3.5.4. Histometric Analysis

Studies utilized cell-based assays and histometric analysis to examine the soft tissue response surrounding anodized abutments. The results consistently showed that there was enhanced connective tissue arrangement and fibroblast activity associated with the anodized titanium abutments. Although one study found there were better early outcomes with slim machined abutments, the overall soft tissue findings associated with anodized abutments were generally favorable [17,23,25,26,30,37,39,48].

## 4. Discussion

The outcomes of this narrative review demonstrate that anodized titanium abutments affect physical, esthetic, and biological properties via surface chemistry, nano and micro topographic changes, and optical masking. However, the strength of evidence varies by outcome and study design, and long-term clinical data remain limited.

### 4.1. Physical Properties

Anodization of titanium abutments modifies physicochemical characteristics primarily by increasing the thickness of the surface TiO_2_ layer and is commonly associated with lower water contact angles and increased hydrophilicity [22,23,26,27,28,29,30,36]. Therefore, higher oxygen signals reported by EDX or related elemental analyses should be interpreted as an apparent increase caused by a thicker oxide layer within the probed analytical depth, rather than as an increase in the oxygen content of the outermost titanium dioxide surface. These effects may be relevant at the transmucosal junction, where protein adsorption and fibroblast behavior contribute to soft tissue integration. Lower contact angles have been correlated with greater fibroblast proliferation, alignment, and focal adhesion formation, which may support more favorable early soft tissue interactions than machined titanium [23,25,26,29]. However, reported hydrophilicity changes should be interpreted in relation to contact-angle testing protocols, because wettability measurements can be affected by surface preparation, storage, contamination, liquid type, and measurement timing. Several studies suggest that anodization alone does not consistently improve fibroblast function unless accompanied by additional micro-roughening [21,23,30]. Voltage effects also appear important, with lower anodization voltages (≤60 V) producing more homogeneous oxide layers and improved hydrophilicity, whereas higher voltages have been associated with micro-cracking and irregular oxide formation [7,23,29]. From a surface property standpoint, voltages at or below 60 V are preferable, as they produce more uniform oxide layers, greater hydrophilicity, and more favorable surface characteristics for soft tissue integration. However, from an esthetic standpoint, higher voltages, particularly in the range of 65 to 75 V, are often necessary to achieve the pink coloration that most closely mimics the peri-implant soft tissue. Clinicians should be aware of this voltage-dependent trade-off when selecting anodization parameters for esthetic zone restorations. Overall, anodization predictably changes wettability and surface chemistry, but these physical changes should be interpreted as contributory rather than determinative of long-term clinical outcomes.

### 4.2. Esthetic Outcomes

Although zirconia abutments demonstrated the most esthetic outcomes, anodized titanium abutments exhibited notable improvements compared to the untreated titanium in scenarios where adequate soft tissue thickness was provided [7,8,14,38,40,42,43,45,50]. These findings suggest that anodization improves titanium’s esthetic outcomes, but zirconia remains the material most consistently associated with superior color outcomes.

The predominant reason for these improved esthetic outcomes is the presence of a thicker oxide layer, which is created during the anodization process. This oxide layer modifies the L*, a*, and b* values by altering the light wave interference. This process minimizes titanium’s inherent gray color, especially in the anodized yellow and pink spectrum [7,12,21,24,46]. Other reasons why anodized titanium abutments display improved esthetics, specifically in soft tissue masking, are changes in the refractive index and decreased contrast between the mucosa and the abutments.

The thickness of the peri-implant soft tissue also contributes to the esthetic outcomes. Multiple studies have demonstrated that soft tissue with ≥2 mm has been shown to decrease the ΔE values regardless of the abutment material [8,40,44,50]. This emphasizes the significance of soft tissue phenotype in establishing appropriate esthetic outcomes. Additionally, other variables such as cement translucency and ceramic thickness also contribute to esthetic outcomes [34,38,47]. This highlights that esthetics are not only reliant on the abutment material alone but on the compound effects of substrate color, soft tissue characteristics, and restorative design limitations. Accordingly, esthetic outcomes should be interpreted as a combined effect of abutment color, soft tissue thickness, and restorative design.

### 4.3. Biological Outcomes

Across the reviewed literature, anodized titanium abutments were frequently associated with improved early soft tissue responses, including fibroblast alignment, adhesion, and viability, and in some protocols reduced bacterial adhesion compared with machined titanium [21,22,23,25,26,27,28,30]. These findings have been linked to higher hydrophilicity, altered surface topography, and changes in surface energy after anodization. However, animal and human clinical studies generally reported small, inconsistent, or non-significant differences between anodized and machined abutments for probing depth, inflammation indices, and marginal bone changes [17,37,39,48,49]. The divergence between laboratory and clinical findings likely reflects the multifactorial nature of peri-implant healing, including abutment geometry (regular versus slim), surgical technique, host factors, and soft tissue phenotype.

Similarly, although several in vitro models reported reduced bacterial adhesion on anodized titanium, clinical studies did not show consistent differences in plaque accumulation or inflammatory parameters [16,21,22,27,28,49]. These observations suggest that anodization may influence early biological events under controlled conditions, but its clinical effect on long-term peri-implant health is limited. Accordingly, anodization is best interpreted as an adjunctive surface modification rather than a primary determinant of peri-implant stability.

### 4.4. Study Limitations

Several limitations should be accounted for when analyzing the findings of this narrative review. First, many of the included studies were in vitro investigations, which may not fully replicate the true biological environment of the peri-implant soft tissue in a clinical study. Second, there was quite a difference with respect to anodization methods, applied voltage, surface preparation, and electrolyte composition amongst the included studies. This may have contributed to variability in the findings. As a result, some findings, especially those based on physicochemical characterization, are descriptive and should be interpreted as mechanistic or surrogate evidence rather than direct proof of clinical superiority. Additionally, there were no standardized outcome measures across studies associated with biological and esthetic outcomes, and long-term clinical studies were limited. The restriction to English-language publications may have introduced language bias. In addition, publication bias cannot be excluded, particularly for laboratory studies where negative findings may be less likely to be published. Because this review used a narrative (qualitative) synthesis, no meta-analysis was performed, and a formal risk-of-bias assessment was not undertaken; therefore, the certainty of evidence for several outcomes cannot be fully quantified. Finally, factors such as soft tissue thickness, abutment geometry, patient variability, and surgical techniques may influence the outcomes independently, regardless of the surface modification. This emphasizes the need for long-term and well-designed clinical studies to determine the effects of anodized titanium abutments.

## 5. Conclusions

Collectively, anodization is a reproducible surface modification that can improve the optical masking of titanium and may support early soft tissue interactions under selected protocols. Anodized titanium abutments are associated with altered oxide-layer characteristics, wettability, and surface topography, which may contribute to enhanced soft tissue interactions. Esthetically, anodization can improve the gray show-through by altering light interference patterns, with pink and yellow anodized abutments demonstrating lower color differences. However, soft tissue thickness, cement opacity, and material thickness also play a contributing role. Anodized titanium abutments are an improvement over machined titanium abutments, although zirconia abutments have shown to provide the most favorable esthetic outcomes. While in vitro studies suggest that anodization may improve early soft tissue responses, in vivo studies show that there are minimal differences found between machined and anodized abutments in probing depth, inflammation, and bone remodeling. Therefore, anodization should be considered primarily an esthetic adjunct rather than a definitive strategy for improving long-term peri-implant biological outcomes or peri-implant soft tissue stability.

## Figures and Tables

**Table 1:** Focus question and search strategy.

Focused Question	For patients with dental implants, what are the effects of anodized titanium abutments compared with other abutment materials on the physical properties of the abutments, peri-implant soft tissue esthetics, and biological properties?
Population	Dental implants [MeSH] OR oral implant OR Dental Implantation, Endosseous [MeSH]
	Implant restoration OR implant supported prosthesis OR implant supported fixed dental prosthesis OR implant supported FDP OR implant supported FPD
Intervention or Exposure	Dental Implant-Abutment Design [MeSH] OR Dental Abutments [MeSH] OR titanium abutment OR implant abutment OR anodized titanium OR anodized titanium abutment OR titanium implant abutment OR titanium alloy abutment OR titanium oxide abutment OR anodized abutment OR anodized pink colored implant
Comparison	(Zirconium Oxide [Supplementary Concept] OR Dental Porcelain [MeSH] OR gold OR Lithia Disilicate [Supplementary Concept] OR Lithium Disilicate) Dental Implant-Abutment Design [MeSH] OR zirconium dioxide abutment OR untreated titanium alloy OR machined titanium abutments
Outcome	Esthetics [MeSH] OR biocompatibility OR Histometric OR Radiographic OR Physical properties
Search combination	(Population terms) AND (abutment and anodization terms) AND (comparison terms) AND (outcome terms)
Language	English
Electronic database	PubMed (MEDLINE)
	Cochrane Central Register of Controlled Trials (CENTRAL)
	EMBASE
	Google Scholar
	greylit.org
Manual journal search	Clinical Oral Implants Research
	International Journal of Oral Maxillofacial Implants
	Clinical Implant Dentistry and Related Research
	Journal of Oral Implantology
	Implant Dentistry
	Journal of Prosthetic Dentistry
	Journal of Prosthodontics
	International Journal of Prosthodontics
	Personal communications on Grey Literature
	Dental material journals
Inclusion criteria	In vitro and in vivo studies
	Published between 2002 and 2025
Exclusion criteria	Methodology or technique papers and review articles Multiple publications on the same dataset Studies without identifiable abutment or transmucosal component information Studies not reporting extractable outcomes aligned with at least one prespecified domain Non-English language publications

**Table 2:** Physical Properties of Anodized Titanium Abutments.

Study (Author, Year)	Study Type	Sample Size	Materials/Groups Compared	Surface Characterization Methods	Key Material Findings
Genova et al., 2022 [21]	In vitro study	4 Surfaces (MAC, Y-SL, Y-DM, Y-MAC)	Machined Ti (MAC), Anodized machined (Y-MAC), Anodized sandblasted/acid-etched (Y-SL), Anodized double etched (Y-DM)	SEM, EDX, Contact angle, surface free energy	EDX analysis was interpreted as showing a higher oxide-related oxygen signal after anodization; anodization also reduced apparent hydrophilicity and mostly eliminated the polar SFE component; roughened anodized surfaces (Y-SL, Y-DM) showed greater hydrophobicity compared with MAC.
Golalipour et al., 2025 [22]	In vitro materials study	16 Ti abutments	Anodized Ti Abutments (63 V) vs. Unanodized Abutments	Profilometer (Ra)**;** FE-SEM	Anodization did not significantly change surface roughness, FE-SEM displayed altered pore morphology with smaller pore diameter.
Gulati et al., 2020 [23]	In vitro materials study	TI Foil Substrates and abutments, multiple per group	Rough-Ti, Micro-Ti, TNP-40, TNP-60, TNP-80	FE-SEM, AFM, XPS/chemistry, WCA	Anodization produced aligned titania nanopores with increased roughness, altered chemistry (TiO_2_ + F), higher hydrophilicity, and mechanically robust nanoporous layer (higher modulus/hardness vs. conventional nanotubes).
Hulka et al., 2022 [24]	In vitro materials study	Ti-Ta alloy specimens (Ti5Ta, Ti15Ta, Ti25Ta, Ti30Ta), multiple per group	Ti–Ta alloys: 5%, 15%, 25%, 30% Ta	Color mapping, microscopy, XRD, oxide mass via Faraday’s Law, current density	Higher voltages produced thicker oxide layers and distinct color changes. Ti-25Ta showed highest hardness and most compact oxide layer; 70 V produced a reddish color with potential esthetic application
Kim et al., 2014 [25]	In vitro laboratory study	240 disks	SM Ti, Co-Cr-Mo, TiN coating, Anodized Ti (AO), Resin-coated Ti, Zirconia	Sa, Sdr (3D optical profilometer), WCA	All surfaces had Sa < 0.5 µm; Zirconia had extremely low roughness (Sa = 0.019 µm) and lowest Sdr; Ti-N, AO, and Zr showed lower contact angles
Kim et al., 2014 [26]	In vitro	60 specimens	SM Ti, Co-Cr-Mo, TiN, Anodized Ti, Resin-coated Ti, Zirconia	Sa, Sq, Sz, Sdr, Sdq, Sal, Str), WCA	Zr showed lowest roughness and WCA, resin had highest roughness and WCA, WCA, Sdr, and Sdq were significantly correlated with fibroblast attachment
De Lima et al., 2024 [27]	In vitro	99 Ti disks	Machined Ti (MACH), Acid-etched Ti (AA), Anodized Ti (AN)	SEM, EDS, Ra, Rq, wettability, water droplet base width	AA had the highest roughness, anodized surfaces were smoother and more hydrophilic, MACH surfaces were the smoothest and least wettable
Liu et al., 2025 [28]	In vitro	117 Ti disks	Unanodized Ti-6Al-7Nb, Gold-anodized, Pink-anodized, each with 3 instrumentation protocols (none, air-polishing, titanium scaling)	Ra, WCA, SEM (surface morphology pre/post instrumentation)	Gold and pink anodization decreased roughness and increased hydrophilicity compared with un-anodized Ti, titanium scaling produced the greatest surface roughness in all groups
Mühl et al., 2022 [29]	In vitro	8 Ti6Al4V disks (4 Anodized, 4 Turned)	Turned Ti6Al4V vs. Yellow-anodized Ti6Al4V	SEM, AFM (Ra), XPS (elemental chemistry + Ti oxidation states), EDS (chemical depth profiles), Dynamic contact angle	Anodization increased TiO_2_ thickness, created a more homogeneous nanostructured surface, raised hydrophilicity, and produced higher Ti^4+^ surface states compared to turned Ti
Mussano et al., 2018 [30]	In vitro	8 Ti-Al-V Cylinders	Machined Ti vs. Pink-anodized Ti (Ti-Al-V alloy cylinders)	SEM, XPS, Contact angle (water and diiodomethane), Surface free energy (Owens–Wendt)	Anodization increased TiO_2_ surface oxide, shifted oxidation state distribution, lowered contact angle, and increased surface free energy compared to machined Ti
Pan et al., 2024 [31]	In vitro	36 specimens	Turned, SB, DAE, SLA, TiN, Anodized	Sa, Sq, Ssk, Sku, Sdr	SLA and DAE showed the highest roughness, turned and TiN surfaces were the smoothest, anodized surfaces displayed intermediate roughness values
Pour et al., 2023 [32]	In vitro	26 abutments	Anodized Ti abutments vs. Non-anodized Ti abutments	SEM, Ra	Anodized abutments showed significantly rougher surfaces and significantly higher tensile bond strength between crown and abutment after thermocycling
Selamet et al., 2025 [33]	In vitro	49 disks (7 per group)	Cast Cr–Co, Laser-sintered Cr–Co, Milled Cr–Co, Milled Ti, Anodized Ti, Stock Ti abutments (control), Milled Zirconia	Ra, Rq, Rmax, SEM (1000× morphology)	Laser-sintered Cr–Co displayed lowest roughness values, milled zirconia had the highest, anodized and stock Ti displayed intermediate roughness. Surface roughness differences significantly influenced microbial retention patterns
Sirawuttipong et al., 2024 [34]	In vitro	48 Ti disks and 28 Zr disks	UT (untreated), AN (anodized), SBAN (sandblasted + anodized), ANSB (anodized + sandblasted), HFAN (HF-etched + anodized), ANHF (anodized + HF-etched)	SEM at ×500 magnification (qualitative surface morphology)	Surface morphology differed visibly among groups- SBAN and ANSB showed rougher, irregular topography, AN produced a smoother, uniform oxide morphology, HF-involved groups showed micro-etching patterns
Squier et al., 2002 [35]	In vitro	80 implants paired with abutments in 4 groups of 20	Standard implant and non-anodized abutment, Standard implant and anodized abutment, synOcta implant and non-anodized abutment, synOcta implant and anodized abutment	Not reported	synOcta and non-anodized abutment had the highest removal torque (37.16 Ncm). Anodization reduced removal torque by ~20%, suggesting a lubricating effect
Traver-Mendez et al., 2023 [36]	In vitro	42 Ti disks	Titanium disks: MAC, AA, AC; Implants: machined vs. anodized body/collar/abutment	SEM, Confocal 3D optical profilometry (Sa, Sz, Ssk, Sdr), EDS	Anodized surfaces showed increased O, Ca, P, Na and altered roughness across implant regions; abutment/collar surfaces smoother (Sa 0.11–0.25 μm) than body regions (Sa 0.95–1.68 μm)
Wang et al., 2023 [37]	In vivo animal	6 beagle dogs, 36 implants	TC4-M (machined Ti-6Al-4V), TC4-Nano (anodized nanotubes), TC4-H/Nano (hydrogenated nanotubes; superhydrophilic)	SEM, AFM (Ra), XPS, EDS, WCA	Nanotube layers formed on TC4-Nano and TC4-H/Nano, hydrogenation increased Ti-OH content; roughness higher for nanotube surfaces; hydrophilicity improved (contact angle ↓ from ~94° → ~38° → ~3.8°).
Wang et al., 2019 [7]	In vitro	153 Ti6Al4V disks and 33 Zr disks	Untreated Ti6Al4V vs. Anodized Ti at 60 V (yellow) vs. 65 V (pink) vs. Zirconia	SEM (grain formation, cracks), AFM (Ra), EDS, WCA	Anodization produced yellow and pink colors, increased grain formation, higher surface roughness, and greater hydrophilicity, anodized Ti altered O/P content, zirconia showed lowest ΔE under gingiva

**Table 3:** Esthetic and Biological Outcomes of Anodized Titanium Abutments.

Study (Author/Year)	Study Type	Sample Size	Abutment/Materials Compared	Esthetic Outcomes Evaluated	Biological Outcomes Evaluated	Key Esthetic/Biological Findings
Bas et al., 2022 [38]	In vitro study	30 zirconia disks, 3 Ti abutment backgrounds (yellow-anodized, gray, pink anodized)	Yellow anodized Ti vs. Gray Ti vs. Pink anodized Ti	Spectrophotometric measurements (CIELab), color measured through Zr substructures of different thickness (0.7, 0.9, 1.1 mm) with pink Ti, gray Ti, yellow Ti, and Zr backgrounds	Not Reported (N/R)	Thickness of Zr significantly affected ΔE (*p* < 0.001), thicker Zr showed lowest ΔE. Abutment color did not affect ΔE
Dib-Zaitum et al., 2022 [39]	In vivo study	10 patients/40 abutments	Slim Anodized (SA) vs. Slim Machined (SM) vs. Regular Machined (RM) vs. Regular Anodized (RA) abutments	N/R	Biological width, epithelial sulcus depth, epithelium height, connective tissue thickness, collagen fiber density, presence of inflammatory cells, vascularization, attached gingiva, ISQ, bone level changes	Slim abutments had better soft tissue response including most epithelial and connective tissue attachment, lower inflammation, and highest collagen. Morphology had greater effect than surface tx while ISQ values and bones levels were similar across all groups.
Farrag et al., 2023 [40]	Prospective in vivo, split-mouth study	28 patients/60 Implants	Unanodized Ti Abutments vs. Pink anodized Ti Abutments	Modified Pink Esthetic Score (mPES) at 1, 12, and 18 month	Recession, probing depth, modified sulcus bleeding index, modified plaque index, modified gingival index	No significant differences or improvement between all groups
Farrag et al., 2022 [41]	In vitro study	40 Ti backgrounds and 20 LD ceramic disks	Un-Anodized Ti under lithium disilicate vs. Yellow anodized Ti	CIE Lab* coordinates, ΔE color difference, evaluation at 1 mm and 2 mm LDS thickness, comparison to control (4 mm LDS)	N/R	Yellow anodized Ti had lower ΔE than unanodized Ti at both thicknesses.
Genova et al., 2022 [21]	In vitro study	4 Surfaces (MAC, Y-SL, Y-DM, Y-MAC)	Machined (MAC), Yellow Anodized Machined (Y-MAC), Anodized Sandblasted/Acid Etched (Y-SL), Anodized Double Etched (Y-DM)	N/R	Bacterial adhesion (S. sanguinis, E. faecalis), protein adsorption, fibroblast adhesion, fibroblast proliferation	All anodized surfaces displayed significant reduction in bacterial adhesion compared to unanodized Ti. Roughened anodized surfaces (Y-SL and Y-DM) had the highest protein adsorption, greater fibroblast adhesion, and increased proliferation. Anodization alone did not enhance fibroblast response (MAC ≈ Y-MAC), but roughness and anodization improved biological performance
Gil et al., 2017 [14]	Prospective RCT	40 Patients	Pink Neck implants/pink anodized Ti abutment vs. Gray implant/gray abutment	CIELAB L*, a*, b* values; ΔE for gray → pink abutment; cervical/middle/incisal measurements	N/R	Pink abutments increased a* (redness) significantly (*p* = 0.01) and produced ΔE ≈ 4.22, above perceptibility threshold, improved soft tissue color regardless of implant neck color
Golalipour et al., 2025 [22]	In vitro study	16 Ti abutments	Anodized vs. Non anodized Ti	N/R	Ra, bacterial adhesion (S. aureus CFU log counts)	Anodization significantly reduced bacterial adhesion (mean log CFU 10.59 vs. 12.90; *p* < 0.001) with no significant change in Ra
Gulati et al., 2020 [23]	In vitro study	TI Foil Substrates and abutments, multiple per group	Micro Ti, Rough Ti, anodized nanoporous Ti (40 V/60 V/80 V)	N/R	Fibroblast proliferation; adhesion and filopodia formation, cell morphology, alignment to nanopores; gene expression (COL-1, COL-3, FN, integrin β1, VEGF, ICAM-1)	Nanoporous surfaces significantly enhanced GF adhesion, proliferation, alignment, and wound-healing gene expression, indicating improved soft tissue integration potential
Hall et al., 2019 [16]	RCT	35 patients	Nanostructured anodized Ti vs. machined Ti Abutments	N/R	Biofilm CFU, proteolytic activity; bleeding index, keratinized mucosa height, plaque index, inflammation index, suppuration, peri-implant probing depth, marginal bone levels, PICF gene expression	No significant differences in biofilm load. Test abutments showed significantly lower bleeding on removal at 6 weeks (*p* = 0.006) and consistently greater keratinized mucosa height at all follow-ups (6 weeks, 6 months, 2 years). No differences in plaque, inflammation, probing depth, or bone levels. Gene expression markers (tPA ↑, Collagen IV ↑ at test sites) suggested improved soft tissue healing
Khorshidi et al., 2024 [42]	In vitro study	19 abutments	Non-Anodized Ti vs. Anodized under 2mm LDS crown	L*, a*, b*, C*, h*, ΔE00 between crown over anodized vs. non-anodized Ti	N/R	Anodized abutments displayed significantly lower ΔE00 (2.26 vs. 2.98) compared to non-anodized Ti with improved color masking and more favorable crown esthetics
Kilinc et al., 2024 [43]	In vitro study	60 abutments (240 total crown/abutment units)	Gold-anodized Ti, Pink-Anodized Ti, Non Anodized Ti, hybrid Ti-Zirconia, PEEK Ti, Composite	L*, a*, b*, C*, h* and ΔE00 measurements of final crown color over each abutment type	N/R	Abutment material significantly affected ΔE00 (*p* < 0.001). Pink-anodized Ti and hybrid abutments produced the lowest color change (ΔE00 ~0.98–1.31), while non-anodized Ti produced the highest (ΔE00 ~2.77). Cement thickness had minimal effect except for VE (0.1 mm caused higher ΔE00). VS masked underlying abutments better than VE
Kim et al., 2014 [25]	In vitro study	240 disks	Co-Cr-Mo, Machined Ti, TiN, Anodized Ti, Resin-coated Ti, Zirconia	N/R	HGF-1 attachment at 1 h; proliferation at days 3 and 7, OD measurements, correlation with surface roughness (Sa), Sdr, and WCA	AO, TiN, and Zr showed the highest fibroblast proliferation at day 7 (≈2× vs. other materials). CCM had the lowest attachment. Surfaces with lower WCA generally supported higher fibroblast response
Kim et al., 2014 [26]	In vitro study	60 specimens	Co-Cr-Mo, TiN, resin coated Ti, Zirconia, SM Ti	N/R	HGF-1 attachment via OD at 590 nm; correlation with Sa, Sq, Sz, Sdr, Sdq, Sal, Str, WCA	Fibroblast attachment highest on SM, TiN, and Zr; lowest on CCM. WCA was the strongest predictor—lower WCA → higher cell attachment. Surfaces with Sa ≤ 0.2 µm showed fibroblast attachment driven primarily by WCA. Developed area ratio (Sdr) and RMS slope (Sdq) also showed secondary correlations
Liu et al., 2025 [28]	In vitro study	117 Ti disks	Gold-Anodized vs. Un- Anodized vs. Pink Anodized Ti and Instrumentation (Ti scaling vs. air polishing)	N/R	Biofilm viability (CFU/mL), biofilm mass (crystal violet absorbance), wettability (hydrophilicity), SEM visualization of biofilm morphology	Gold- and pink-anodized surfaces showed reduced biofilm viability compared with un-anodized Ti, air-polishing significantly decreased biofilm viability and mass for all groups, titanium scaling increased roughness and biofilm formation, hydrophilicity moderately correlated with reduced CFU
Martinez-Rus et al., 2017 [8]	Prospective RCT	20 Patients	Ti, Gold -Anodized Ti, Pink Anodized Ti, Zirconia Abutments	CIELAB L*, a*, b*, ΔE of peri-implant soft tissue and coronal crown, effect of soft tissue thickness on ΔE	N/R	Zr and gold-anodized Ti displayed the lowest soft tissue ΔE, titanium and pink-anodized Ti displayed highest ΔE, soft tissue thickness negatively correlated with ΔE for Ti and pink-anodized abutments
Mussano et al., 2018 [30]	In vitro study	8 Ti-Al-V CYLINDERS	Machined Ti vs. Anodized Ti	N/R	Fibroblast and epithelial cell adhesion, cell viability at 24–72 h, focal adhesion density, cell morphology, cytoskeletal organization, wettability	Anodized Ti showed significantly increased fibroblast and epithelial adhesion and viability vs. machined Ti, higher focal adhesion density and more favorable early soft tissue cell response
Pan et al., 2024 [31]	In vitro study	36 specimens	Ti, Ti ALD, Ti AO, ZrO_2_	N/R	Single-species and mixed-species bacterial adhesion assays (*S. mutans, S. aureus, P. gingivalis*); CFU counts; MTT metabolic activity; SEM biofilm morphology	ALD-TiO_2_ reduced bacterial adhesion by ≥50% vs. uncoated Ti and performed similarly to ZrO_2_, AO-TiO_2_ showed no antibacterial advantage, ALD maintained smooth anatase-phase coating and demonstrated the lowest biofilm activity across assays
Selamet et al., 2025 [33]	In vitro study	49 disks (7 per group)	Milled Cr-Co, Cast Cr-Co, Laser Sintered Cr-Co, Milled Ti, Stock Ti, Milled Zirconia, Anodized Milled Ti	N/R	Biofilm formation by *Streptococcus mutans* and *Candida albicans* at 8 h and 24 h, CFU (log_10_) counts	Milled zirconia showed the highest S. mutans colonization (log_10_ ≈ 5.87 at 24 h); cast and milled Cr–Co showed the lowest bacterial counts, *C. albicans* colonization was highest on stock Ti and lowest on anodized milled Ti. Roughness did not fully predict biofilm formation, indicating material composition also influences microbial adhesion
Sen et al., 2025 [44]	In vitro study	50 simulated gingival specimens	Zirconia Abutments under 5 gingival colors (1.0 mm and 2.0 mm thickness vs. Ti	CIELAB L*, a*, b*; ΔE*ab and ΔE00 color differences, comparison with perceptibility and acceptability thresholds	N/R	Titanium abutments with lighter gingival colors (light pink, orange) at 1.0 mm showed moderately unacceptable ΔE (above AT). Zirconia abutments yielded ΔE below AT for all gingival colors and both thicknesses. Increasing gingival thickness (2.0 mm) significantly reduced ΔE in LP, DP, and Or groups with Ti abutments. Abutment material significantly affected ΔE00 at 1.0 mm but not at 2.0 mm.
Senkiw et al., 2024 [45]	In vitro study	60 Zr crowns and 6 Ti Abutments	3Y vs. 4Y vs. 5Y Zr over Ti anodized at 0 V, 11 V, 31 V, 54 V, 64 V, 76 V	Spectrophotometric L*, a*, b* values and ΔE00 for each zirconia material over each anodization color	N/R	Significant differences in ΔE00 across anodization voltages (*p* < 0.0001). Blue (31 V) had the highest ΔE00 for all zirconia types. Lowest ΔE00 was unanodized Ti for 3Y and yellow (64 V) for 4Y and 5Y. Yttria content (3Y vs. 4Y vs. 5Y) showed no significant effect on ΔE00. Anodization voltage was the dominant factor influencing final crown shade
Seyidaliyeva et al., 2022 [46]	In vitro study	192 TI alloy specimens	Polished and Anodized (PA) vs. Sandblasted (S) vs. Sandblasted and Anodized (SA) vs. Polished Etched Anodized (PEA)	CIELAB L*, a*, b* values; ΔE00 between surface treatment groups (optical color of titanium only, not soft tissue or crown color)	N/R	Anodizing produced the brightest and most chromatic colors (highest L*, a*, b*). Sandblasting produced the darkest, grayest, and most predictable color with the least variability. All groups showed large ΔE00 differences (>16), indicating strong optical changes from surface treatment alone.
Seyidaliyeva et al., 2024 [47]	In vitro study	192 Ti Substrates and 192 Zr disks	Titanium (Polished, Etched, Anodized, Sandblasted) and Zr Thickness (0.7/1.0 mm) cement type (translucent vs. opaque)	CIELAB L*, a*, b* values of zirconia and titanium, ΔE00 (zirconia vs. total assembly), effect of substrate, zirconia thickness, and cement opacity on final zirconia color	N/R	Opaque cement masked the titanium substrate (ΔE00 ≈ 5.5–6.2) with minimal color shifts. Translucent cement caused large color changes (ΔE00 up to 11.7), making zirconia darker, more reddish, and more yellowish; zirconia thickness had only a minor effect. Substrate color strongly affected outcomes only with translucent cement
Sirawuttipong et al., 2024 [34]	In vitro study	48 Ti disks and 28 Zr disks	Untreated Ti vs. Anodized Ti vs. ANSB vs. SBAN vs. ANHF vs. HFAN	CIELAB L*, a*, b*; ΔE* relative to perceptibility/acceptability thresholds for each zirconia thickness–cement–titanium surface combination	N/R	Zirconia thickness, cement shade, and Ti surface treatment significantly affected ΔE*. Only 2.5 mm HT zirconia + clear cement produced ΔE* < 2.7 (clinically acceptable) over AN, UT, or SBAN surfaces. Thinner zirconia and opaque cement resulted in unacceptable color differences, regardless of surface treatment.
Susin et al., 2019 [17]	In vivo animal study	12 mini-pigs, 72 Implants	Machined Ti Abutments vs. Anodized Abutments	N/R	Histologic soft tissue healing (inflammation scores, epithelium length, mucosal height, junctional epithelium position), peri-implant bone parameters (crestal bone level, first BIC, %BIC; bone density)	No significant differences between anodized and machined abutments for inflammation, epithelial length, mucosal height, BIC, or bone density. Radiographically, 6-week crestal bone loss was lower for anodized abutments (0.8 mm vs. 2.1 mm; *p* = 0.046), but no differences at 13 weeks
Susin et al., 2019 [48]	In vivo animal study	24 Yucatan Mini-Pigs, 96 implants	Commercially available anodized implant/machined abutment vs. gradually anodized implant/anodized abutment with a protective layer	N/R	MicroCT and histology, inflammation scores; mucosal height; epithelium length; epithelium–platform distance, buccal/lingual crestal bone levels, BIC, trabecular thickness/spacing, BDWT/BDOT	No significant differences in soft tissue inflammation, epithelial length, BIC, bone density, or crestal bone levels at any timepoint. A small early mucosal-height difference at 3 weeks was transient. Trabecular spacing was borderline better in the test group at 3 weeks. The gradually anodized system showed equivalent biological performance to the predicate anodized implant/machined abutment
Traver-Mendez et al., 2023 [36]	In vitro study	42 Ti disks,	Anodized abutment-type Ti vs. Anodized collar type Ti vs. Machined Ti	N/R	HaCat + BM-MSC adhesion (SEM), proliferation at days 1, 3, 7 (resazurin assay), qualitative cell morphology and spreading	Both cell types showed significant proliferation over time, with no statistical differences among machined vs. anodized surfaces. SEM showed good adhesion on all surfaces, with enhanced HaCat attachment on anodized collars and BM-MSC adhesion on both anodized and machined abutments. Anodization introduced Ca, P, Na, and increased surface complexity. Overall, positive biocompatibility but no superiority vs. machined.
Vazouras et al., 2022 [49]	Prospective RCT	25 Patients	Pink- Anodized Ti vs. Gray Anodized Ti vs. Hybrid Zr Abutments	Peri-implant mucosal ΔE (CIELAB) vs. contralateral tooth, Pink Esthetic Score (PES), Patient satisfaction (VAS)	N/R	Zirconia showed lowest ΔE, followed by pink anodized, then gray Ti. PES was significantly higher for zirconia and pink anodized only in thin biotype. At 1 year, no difference in patient satisfaction between Zr and pink anodized
Wang et al., 2023 [37]	In vivo	6 beagle dogs, 36 implants	Machined Ti-6Al-4V vs. Anodized nanotubes vs. super-hydrophilic nanotubes	N/R	Histology and histometry of sulcular epithelium length (SE), junctional epithelium (JE), connective tissue (CT) length, biological width (BW), collagen fiber orientation (SHG microscopy), inflammatory cell levels	All abutments showed similar soft tissue dimensions at both timepoints. SHG imaging showed partially perpendicular collagen fibers adjacent to the TC4-H/Nano surface vs. parallel in TC4-M and TC4-Nano. Overall, nanotubular/superhydrophilic surfaces demonstrated comparable soft tissue healing with potential improvement in CT functional attachment.
Wang et al., 2019 [7]	In vitro study	153 Ti6Al4V disks and 33 Zr disks	Anodized Ti (65 V Pink and 60 V Yellow) vs. untreated Ti vs. Zr Disks	Gingival color change (ΔE) under 1 mm pig gingiva for pink and yellow anodized Ti6Al4V vs. untreated Ti and zirconia	HGF morphology (SEM), proliferation (CCK-8), viability (live/dead stain), contact angle and surface roughness effects on fibroblast behavior	Pink and yellow anodized Ti showed significantly lower ΔE than untreated Ti but higher than zirconia, anodization increased roughness and hydrophilicity, HGFs showed normal morphology, adhesion, and viability similar to untreated Ti, zirconia had highest proliferation and lowest cell death
Wang et al., 2021 [50]	Clinical study	11 participants	Gold-Anodized Ti vs. Un-Anodized Ti vs. Pink-Anodized Ti vs. Zr	Peri-implant mucosal ΔE (CIE Lab)* vs. contralateral natural gingiva; evaluation of L*, a*, b* values, assessment of soft tissue thickness	Gingival thickness (transgingival probing with endodontic file)	All abutments produced ΔE > 3.7 (visible discoloration). ΔE ranking (best → worst): zirconia (6.81), pink Ti (7.63), gold Ti (7.90), unanodized Ti (8.74). Pink and gold anodized Ti improved esthetics vs. uncolored Ti, but zirconia had the best overall match. Mean gingival thickness was 2.41 ± 0.52 mm.

## Data Availability

Data supporting the findings of this study are available from the corresponding author upon reasonable request.

## References

[1] Dede D.O., Armaganci A., Ceylan G., Celik E., Cankaya S., Yilmaz B. (2016). Influence of implant abutment material on the color of different ceramic crown systems. J. Prosthet. Dent..

[2] Aslan M.A., Ural C., Arici S. (2016). Investigation of the effect of titanium alloy surface coating with different techniques on titanium-porcelain bonding. J. Prosthet. Dent..

[3] Kim A., Campbell S.D., Viana M.A., Knoernschild K.L. (2016). Abutment Material Effect on Peri-implant Soft Tissue Color and Perceived Esthetics. J. Prosthodont..

[4] Ajlouni K., Elshahawy W., Ajlouni R., Sadakah A. (2018). Color masking measurement for ceramic coating of titanium used for dental implants. J. Prosthet. Dent..

[5] Diamanti M.V., Del Curto B., Pedeferri M. (2011). Anodic oxidation of titanium: From technical aspects to biomedical applications. J. Appl. Biomater. Biomech..

[6] Mazzarolo A., Curioni M., Vicenzo A., Skeldon P., Thompson G.E. (2012). Anodic growth of titanium oxide: Electrochemical behaviour and morphological evolution. Electrochim. Acta.

[7] Wang T., Wang L., Lu Q., Fan Z. (2019). Changes in the esthetic, physical, and biological properties of a titanium alloy abutment treated by anodic oxidation. J. Prosthet. Dent..

[8] Martínez-Rus F., Prieto M., Salido M.P., Madrigal C., Özcan M., Pradíes G. (2017). A Clinical Study Assessing the Influence of Anodized Titanium and Zirconium Dioxide Abutments and Peri-implant Soft Tissue Thickness on the Optical Outcome of Implant-Supported Lithium Disilicate Single Crowns. Int. J. Oral Maxillofac. Implant..

[9] Napoli G. (2018). Colouring titanium alloys by anodic oxidation. Metalurgija.

[10] Roberge P.R. (2019). Handbook of Corrosion Engineering.

[11] Paschalis E.I., Chodosh J., Spurr-Michaud S., Cruzat A., Tauber A., Behlau I., Gipson I., Dohlman C.H. (2013). In vitro and in vivo assessment of titanium surface modification for coloring the backplate of the Boston keratoprosthesis. Investig. Ophthalmol. Vis. Sci..

[12] Wadhwani C., Brindis M., Kattadiyil M.T., O’Brien R., Chung K.H. (2018). Colorizing titanium-6aluminum-4vanadium alloy using electrochemical anodization: Developing a color chart. J. Prosthet. Dent..

[13] Wadhwani C.P.K., Schoenbaum T., King K.E., Chung K.H. (2018). Techniques to Optimize Color Esthetics, Bonding, and Peri-implant Tissue Health With Titanium Implant Abutments. Compend. Contin. Educ. Dent..

[14] Gil M.S., Ishikawa-Nagai S., Elani H.W., Da Silva J.D., Kim D.M., Tarnow D., Schulze-Späte U., Bittner N. (2017). A prospective clinical trial to assess the optical efficacy of pink neck implants and pink abutments on soft tissue esthetics. J. Esthet. Restor. Dent..

[15] Huang J.W., Chen W.C., Huang T.K., Fu P.S., Lai P.L., Tsai C.F., Hung C.C. (2011). Using a spectrophotometric study of human gingival colour distribution to develop a shade guide. J. Dent..

[16] Hall J., Neilands J., Davies J.R., Ekestubbe A., Friberg B. (2019). A randomized, controlled, clinical study on a new titanium oxide abutment surface for improved healing and soft tissue health. Clin. Implant Dent. Relat. Res..

[17] Susin C., Finger Stadler A., Fiorini T., de Sousa Rabelo M., Ramos U.D., Schüpbach P. (2019). Safety and efficacy of a novel anodized abutment on soft tissue healing in Yucatan mini-pigs. Clin. Implant Dent. Relat. Res..

[18] Yetim A. (2010). Investigation of wear behavior of titanium oxide films, produced by anodic oxidation, on commercially pure titanium in vacuum conditions. Surf. Coat. Technol..

[19] Dong H., Bell T. (2000). Enhanced wear resistance of titanium surfaces by a new thermal oxidation treatment. Wear.

[20] Kahar M.S., Macwan A., Oza M.R., Oza V., Shah S. (2013). Characterization and Corrosion Study of Titanium Anodized Film Developed in KOH Bath. J. Eng. Res. Appl..

[21] Genova T., Chinigò G., Munaron L., Rivolo P., Luganini A., Gribaudo G., Cavagnetto D., Mandracci P., Mussano F. (2022). Bacterial and Cellular Response to Yellow-Shaded Surface Modifications for Dental Implant Abutments. Biomolecules.

[22] Golalipour S., Jalalian E., Koosha S., Khorshidi S., Torshabi M., Sayyari M. (2025). In vitro effect of anodization on surface roughness and bacterial adhesion to titanium abutments. J. Prosthet. Dent..

[23] Gulati K., Moon H.J., Kumar P.T.S., Han P., Ivanovski S. (2020). Anodized anisotropic titanium surfaces for enhanced guidance of gingival fibroblasts. Mater. Sci. Eng. C Mater. Biol. Appl..

[24] Hulka I., Florido-Suarez N.R., Mirza-Rosca J.C., Saceleanu A. (2022). Ti–Ta dental alloys and a way to improve gingival aesthetic in contact with the implant. Mater. Chem. Phys..

[25] Kim Y.S., Ko Y., Kye S.B., Yang S.M. (2014). Human gingival fibroblast (HGF-1) attachment and proliferation on several abutment materials with various colors. Int. J. Oral Maxillofac. Implant..

[26] Kim Y.S., Shin S.Y., Moon S.K., Yang S.M. (2015). Surface properties correlated with the human gingival fibroblasts attachment on various materials for implant abutments: A multiple regression analysis. Acta Odontol. Scand..

[27] Lima J.H.C., Robbs P.C.M., Tude E.M.O., De Aza P.N., Costa E.M.D., Scarano A., Prados-Frutos J.C., Fernandes G.V.O., Gehrke S.A. (2024). Fibroblasts and osteoblasts behavior after contact with different titanium surfaces used as implant abutment: An in vitro experimental study. Heliyon.

[28] Liu W., Gregory R.L., Yang C.C., Hamada Y., Lin W.S. (2025). The effects of anodization and instrumentation on titanium abutment surface characteristics and biofilm formation. J. Prosthodont..

[29] Mühl A., Szabó P., Krafcsik O., Aigner Z., Kopniczky J., Ákos Nagy Marada G., Turzó K. (2022). Comparison of surface aspects of turned and anodized titanium dental implant, or abutment material for an optimal soft tissue integration. Heliyon.

[30] Mussano F., Genova T., Laurenti M., Zicola E., Munaron L., Rivolo P., Mandracci P., Carossa S. (2018). Early Response of Fibroblasts and Epithelial Cells to Pink-Shaded Anodized Dental Implant Abutments: An In Vitro Study. Int. J. Oral Maxillofac. Implant..

[31] Pan Y., Cao L., Chen L., Gao L., Wei X., Lin H., Jiang L., Wang Y., Cheng H. (2024). Enhanced Bacterial and Biofilm Adhesion Resistance of ALD Nano-TiO_2_ Coatings Compared to AO Coatings on Titanium Abutments. Int. J. Nanomed..

[32] Pour H.K., Ansari H., Tehrani A.M. (2023). In vitro effect of anodization of titanium abutments on their tensile bond strength to implant-supported lithium disilicate all-ceramic crowns. Dent. Res. J..

[33] Selamet S.M., Kumbuloglu O., Sahiner A., Ozdemir G. (2025). Assessment of surface roughness and microbiological retention on custom abutments produced by different techniques and materials. Eur. J. Oral Sci..

[34] Sirawuttipong C., Palanuwech M. (2024). Effect of Zirconia Thickness, Cement Color, and Titanium Implant Abutment Surface Treatment Type on the Esthetic Outcomes of High-Translucency Monolithic Zirconia. Int. J. Oral Maxillofac. Implant..

[35] Squier R.S., Psoter W.J., Taylor T.D. (2002). Removal torques of conical, tapered implant abutments: The effects of anodization and reduction of surface area. Int. J. Oral Maxillofac. Implant..

[36] Traver-Méndez V., Camps-Font O., Ventura F., Nicolau-Sansó M.A., Subirà-Pifarré C., Figueiredo R., Valmaseda-Castellón E. (2023). In Vitro Characterization of an Anodized Surface of a Dental Implant Collar and Dental Abutment on Peri-Implant Cellular Response. Materials.

[37] Wang C., Wang X., Lu R., Cao X., Yuan D., Chen S. (2023). Influence of surface nanotopography and wettability on early phases of peri-implant soft tissue healing: An in-vivo study in dogs. BMC Oral Health.

[38] Bas B.B., Cakan U. (2022). Evaluation of the effect of anodization-colored titanium abutments and zirconia substructure thickness on zirconia substructure color: An In vitro study. Niger. J. Clin. Pract..

[39] Dib-Zaitum I., Guadilla-González Y., Flores-Fraile J., Dib-Zakkour J., Benito-Garzón L., Montero J. (2022). Effect Morphology and Surface Treatment of the Abutments of Dental Implants on the Dimension and Health of Peri-Implant Biological Space. Materials.

[40] Farrag K.M., Khamis M.M. (2023). Effect of anodized titanium abutment collars on peri-implant soft tissue: A split-mouth clinical study. J. Prosthet. Dent..

[41] Farrag K., Bakry S., Aly Y. (2022). Effect of anodic oxidation of titanium substrate on the shade of lithium disilicate ceramic with different thicknesses (in vitro study). Alex. Dent. J..

[42] Khorshidi S., Zarbakhsh A., Lawaf S., Golalipour S., Sayyari M., Nahavandi A.M. (2024). In Vitro Effect of Anodization of Titanium Abutments on Color Parameters and Color Difference of Lithium Disilicate All-Ceramic Crowns. Clin. Exp. Dent. Res..

[43] Kilinc H., Sanal F.A. (2024). Influence of Abutment Material, Cement Thickness, and Crown Type on the Final Color of Implant-Supported Restorations. Int. J. Prosthodont..

[44] Şen N., Şermet I.B. (2025). Influence of Gingival Color and Abutment Material on the Final Color of Peri-Implant Soft Tissue: An In Vitro Analysis. J. Oral Implantol..

[45] Senkiw K. (2024). Shade outcome of 3Y, 4Y, 5Y monolithic zirconia on colored anodized titanium. Dent. Rev..

[46] Seyidaliyeva A., Rues S., Evagorou Z., Hassel A.J., Büsch C., Rammelsberg P., Zenthöfer A. (2022). Predictability and outcome of titanium color after different surface modifications and anodic oxidation. Dent. Mater. J..

[47] Seyidaliyeva A., Zenthöfer A., Rues S. (2024). Evaluation of the Color of Zirconia in Different Substrates of Osseointegrated Implants, Thickness of Materials and Types of Resin Cements. Int. J. Dent..

[48] Susin C., Finger Stadler A., Musskopf M.L., de Sousa Rabelo M., Ramos U.D., Fiorini T. (2019). Safety and efficacy of a novel, gradually anodized dental implant surface: A study in Yucatan mini pigs. Clin. Implant Dent. Relat. Res..

[49] Vazouras K., Gholami H., Margvelashvili-Malament M., Kim Y.J., Finkelman M., Weber H.P. (2022). An Esthetic Evaluation of Different Abutment Materials in the Anterior Maxilla: A Randomized Controlled Clinical Trial Using a Crossover Design. J. Prosthodont..

[50] Wang T., Wang L., Lu Q., Fan Z. (2021). Influence of anodized titanium abutments on the esthetics of the peri-implant soft tissue: A clinical study. J. Prosthet. Dent..

